# Quantification of Lycopene, β-Carotene, and Total Soluble Solids in Intact Red-Flesh Watermelon (*Citrullus lanatus*) Using On-Line Near-Infrared Spectroscopy

**DOI:** 10.3390/s17040746

**Published:** 2017-04-11

**Authors:** Elena Tamburini, Stefania Costa, Irene Rugiero, Paola Pedrini, Maria Gabriella Marchetti

**Affiliations:** Department of Life Science and Biotechnology, University of Ferrara, Via L. Borsari 46, Ferrara 44121, Italy; stefania.costa@unife.it (S.C.); irene.rugiero@unife.it (I.R.); pdp@unife.it (P.P.); mhm@unife.it (M.G.M.)

**Keywords:** watermelon, lycopene, β-carotene, carotenoids, total soluble solid (TSS), Near Infrared Spectroscopy

## Abstract

A great interest has recently been focused on lycopene and β-carotene, because of their antioxidant action in the organism. Red-flesh watermelon is one of the main sources of lycopene as the most abundant carotenoid. The use of near-infrared spectroscopy (NIRS) in post-harvesting has permitted us to rapidly quantify lycopene, β-carotene, and total soluble solids (TSS) on single intact fruits. Watermelons, harvested in 2013–2015, were submitted to near-infrared (NIR) radiation while being transported along a conveyor belt system, stationary and in movement, and at different positions on the belt. Eight hundred spectra from 100 samples were collected as calibration set in the 900–1700 nm interval. Calibration models were performed using partial least squares (PLS) regression on pre-treated spectra (derivatives and SNV) in the ranges 2.65–151.75 mg/kg (lycopene), 0.19–9.39 mg/kg (β-carotene), and 5.3%–13.7% (TSS). External validation was carried out with 35 new samples and on 35 spectra. The PLS models for intact watermelon could predict lycopene with R^2^ = 0.877 and SECV = 15.68 mg/kg, β-carotene with R^2^ = 0.822 and SECV = 0.81 mg/kg, and TSS with R^2^ = 0.836 and SECV = 0.8%. External validation has confirmed predictive ability with R^2^ = 0.805 and RMSEP = 16.19 mg/kg for lycopene, R2 = 0.737 and RMSEP = 0.96 mg/kg for β-carotene, and R^2^ = 0.707 and RMSEP = 1.4% for TSS. The results allow for the market valorization of fruits.

## 1. Introduction

Today, fresh food intake is becoming an even greater matter of health and well-being, rather than solely a concern of personal predilections or taste, due to the presence, especially in fruits, of several bioactive compounds, including carotenoids, flavonoids, phenolic compounds, and vitamins [1]. Watermelon (*Citrullus lanatus*) is a member of the Cucurbitaceae family of gourds and is related to the cucumber, squash, and pumpkin [2]. Watermelon flesh is about 91% water by weight, and is a rich source of bioavailable compounds including lycopene, β-carotene and other carotenoids, citrulline, and vitamins A and C [3]. In particular, lycopene and β-carotene are lipophilic natural carotenoids that have been well studied as antioxidant agents against oxygen free radicals, concurring to reduce the overall oxidative stress of the organism [4]. Moreover, lycopene and β-carotene daily consumption is reported to assure significant positive effects on human health [5]. For example, lycopene has been shown to reduce prostate cancer incidence [6] and to prevent Alzheimer’s disease pathogenesis [7]. In addition, high levels of circulating lycopene have been associated with cholesterol serum levels decreasing [8,9] and have been shown to be effective against several common diseases including cancer [10], pancreatitis [11], gastritis [12], atherosclerosis [13], and heart disease [14]. Although most studies for the development of functional foods have focused on tomato as the richest source of lycopene, lycopene from watermelon has lately received much attention because of its proven benefits for human health [15,16]. Watermelon is found to contain more lycopene than most other foods [17]. As reported by Edwards et al. [18], the lycopene content of watermelon is about 50 mg/100 g fresh watermelon, on average higher than the lycopene contained in fresh tomato (30 mg/100 g). Moreover, heating does not seem to be required for the absorption of lycopene from watermelon compared to tomato. Fresh watermelons are also rich in other bioactive molecules, such as lutein and citrulline, that exert a synergistic action with lycopene [19].

Several methods for measuring carotenoid concentration in food have been reported in the literature. The majority of them are based on colorimetric or chromatographic assays after organic solvent extraction [20]. However, these methods are destructive, laborious, and time-consuming, requiring the preparation of samples with hazardous and expensive reagents. The development of a fast, non-destructive, and real-time analytical assay is strongly desirable [21]. Recently, various spectroscopic methods, including hyperspectral reflectance imaging [22], ATR spectroscopy [23], Raman chemical imaging [24], and magnetic resonance imaging [25], have been developed, but there are principal limitations to their diffusion at the industrial level, such as the fact that they are very expensive and that they cannot be used on-line or in-line. As an alternative, near-infrared spectroscopy (NIRS) has been proposed. NIRS is a well-established and largely applied technique for measuring quality parameters in food [26]. NIRS is a non-destructive method, which requires a minimum of sample treatment before analysis. It can be applied to measure several parameters at the same time and can be used for a wide range of products, allowing for a real-time decision based on NIRS results [27,28,29]. The use of NIRS for lycopene determination has been widely reported in tomato pulp and skin [30,31] and tomato juice [32]. In recent years, the NIRS method has been successfully developed for non-destructive analysis of internal watermelon quality, such as sugar content [33] and total soluble solid (TSS) content [34]. However, to the best of the authors’ knowledge, so far no published data has referred to NIRS for the direct determination of carotenoid content in intact watermelon.

Therefore, we describe in this paper the development of an NIRS in-line application in the post-harvesting stage as a direct and non-destructive method to determine lycopene and β-carotene content in single intact fruits, while simultaneously estimating the Brix degree. The results of this study have been applied to the market valorization of the analyzed watermelons; each of them has been commercialized for its unique lycopene, β-carotene, and TSS content, immediately visible to consumers through a label on the fruit skin.

## 2. Materials and Methods

### 2.1. Watermelon Fruit Sampling

The watermelon *Citrullus lanatus*, cv. MINIROSSA™, selected for having high carotenoid content, has been used for all the experimental work. MINIROSSA™ is characterized by a small size (diameter, 100–150 mm) and a very thin, striped green rind (<0.5 mm). All fruits were grown in an open field in the same area (the northeast of Italy) under a Mediterranean climate during 3 campaigns from 2013 to 2015. Fruits were harvested on multiple harvest dates from late June to mid-September of every year at dates according to the maturity stage of each one. No standardized index for watermelon ripening is available, so field ripeness was usually judged by skilled inspectors who slap the surface of the watermelon and determine ripeness on factors such as the pitch and tone of the sound produced, giving an unavoidable margin of error. During 2013, 40 samples were picked up corresponding to 100% grade of ripening, whilst in 2014 60 samples were picked, 12 for each of the 5 ripening stage: 50% (completely unripe), 80% (almost ripe), 100% (ripe), 110% (just overripe), and 130% (completely overripe). In 2015, another 35 fruits were sampled at randomly different grades of ripening from the field. During the 2013 campaign, samples were transported to our laboratory within a day after harvest and processed with a near-infrared (NIR) instrument in a few hours. During the 2014 campaign, each fruit was processed in the farm, and submitted to NIR radiation directly on the sorting machine along a conveyor belt system, which transports fruits from collecting containers to the auto-weighing fruit selector machine.

Reference analysis were performed 2 days later on refrigerated (at a temperature of 4 °C) samples of flesh. Lycopene concentration varied from 2.65 and 151.75 mg/kg of fresh pulp, β-carotene concentration from 0.19 to 9.39 mg/kg of fresh pulp, and TSS concentration from 5.3 to 13.7%. In 35 new samples used for external validation, concentration intervals of 7.0–141.2 mg/kg, 2.00–11.6 mg/kg, and 8.8–13.2% for lycopene, β-carotene, and TTS, respectively, were found.

### 2.2. Spectral Measurements

The reflectance spectra of intact watermelon were collected with a NIR On-Line^®^ X-One (Buchi, Flawil, Switzerland), equipped with a diode array detector (DAD) and a tungsten-halogen dual lamp. The selected wavelength range used was from 900 to 1700 nm, with a measuring time of 10 ms and a reading every 10 nm, for a total of 400 reflectance readings per sampled area. Whole watermelons were placed on the belt in such a way that the fruit rind passed at a distance of 40 mm from the light window. In the 2013 sample campaign, each fruit was submitted to the light beam and positioned on the stationary belt at two different sides, obtaining two spectra for each fruit, for a total of 80 spectra. In 2014, each fruit was sampled on the moving belt at different speed rates (2100, 2400, and 2700 rpm) and, for each speed rate, fruits were positioned at 4 different sides, in order to simulate when fruits randomly fall down on the belt from the collecting containers, obtaining 12 spectra for each fruit, for a total of 720 spectra. A data set of 800 spectra was collected throughout the two campaigns.

Finally, in 2015, for each of the 35 new fruits analyzed, one spectrum was collected while passing on the belt in a random position, as should happen in a routine analysis, so a total of 35 spectra were acquired.

### 2.3. Data Analysis and Chemometrics

Before calibration, raw spectra were pre-processed by applying mathematical pretreatments, i.e., standard normal variate (SNV) and first derivative. SNV is used to minimize slope shifts and multiplicative interferences due to scattering, particle size, and a possible change in light distance [35]. The first derivative in general adjusts the baseline drifts and enhances small spectral differences, by means of Savitzky–Golay polynomial fitting, where the data within a moving window are fitted by a polynomial of a given degree to generate a differential of a chosen degree [36]. A 7-point window and a second-order polynomial were used here for smoothing. Qualitative analysis was based on principal component analysis (PCA) to evaluate the presence of some data grouping and, as a result, to identify separated clusters in the principal components’ (PCs’) plot [37]. Outliers were detected using Mahalanobis distance criterion [38].

Partial least squares (PLS) regression was computed on the calibration set of the 800 pre-treated spectra using SX-Plus™ software (Buchi, Flawil, Switzerland). Model performance was determined using the cross-validation approach, making a random leave-out representing 30% of the samples. Statistics were evaluated by means of a coefficient of determination (R^2^), a standard error of calibration (SEC), a standard error of cross-validation (SECV), and a relative prediction deviation (RPD). External validation was performed by comparing NIR predicted data and reference data in Microsoft Excel^®^ 2010 and by calculating the coefficient of determination (R^2^) and the root mean square error of prediction (RMSEP) as reported by Martens and Naes [39].

### 2.4. Reference Assays

Four or five portions of fresh pulp were taken close to the rind and from the heart area (between the locule and the fruit center) of each watermelon, and mixed together in order to obtain homogeneous samples to submit for reference analysis. 

Carotenoid concentration in watermelon was determined after organosolv extraction (2 g of fresh pulp in 20 mL of chloroform), paying attention to work in the dark and protected from air oxygen. The solvent was evaporated, and samples were re-dissolved in 3 mL of chloroform and submitted for analysis by means of reversed phase HPLC equipped with a MD-2010Plus photo-DAD (Jasco, Tokyo, Japan) [40]. The column was a YMC C30 (250 mm × 4.6 mm, 5 µm), for carotenoid analysis and eluents: (A) methanol/methilterbutilether/water (81/15/4) and (B) methanol/methilterbutilether/water (6/90/4), with a gradient from 1% to 100% of Solution B in 50 min and a mobile phase flow of 1 mL/min. Peak detection was assessed at 450 nm by means of an UV detector. TSS content was obtained as a refractometric Brix Degree measurement.

## 3. Results and Discussion

### 3.1. NIR Methods Development

**Figure 1:**
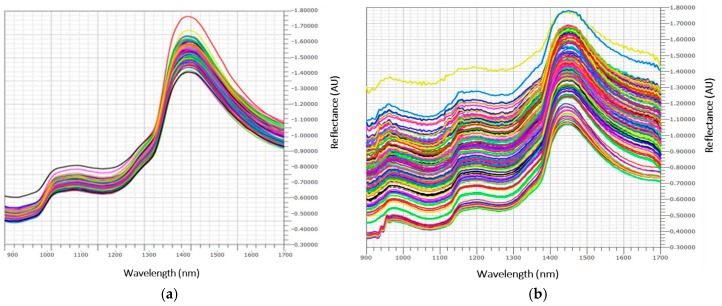
Original reflectance spectra of intact watermelons collected in 2013 (**a**) and 2014 (**b**).

Original reflectance spectra are shown in Figure 1a,b, corresponding to 2013 and 2014 sample campaigns, respectively.

**Figure 2:**
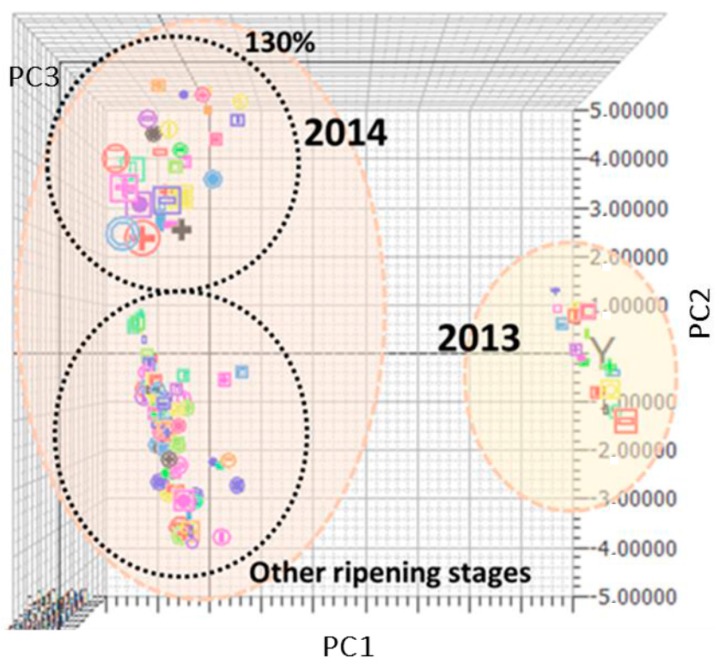
First three principal component (PC) plots derived from component analysis (CA) on calibration set samples. Two clusters can be evidenced: a first (**light pink filling**), which highlighted the year of sampling, and a second (**black dots**), which highlighted the stages of ripening.

Baseline offset of the spectra collected in 2014 could be due to the effect of movement at different speed rates, whilst in 2013 spectra were collected in stationary mode. In the spectra of fresh fruit, the signal of water at 1460 nm, due to the first O–H overtone and O–H combination band, is usually prevailing [41]. Moreover, vegetal tissue components, such as superficial waxes, can cause significant interference on the spectral signal of fresh fruits, which is always difficult to be visually interpreted. Nevertheless, after mathematical pretreatments (data not shown), it was more evident that the wavelength regions of the spectra that show the highest correlation are in the ranges 900–1050 and 1400–1500 nm, corresponding to the 2nd and 1st overtone of the O–H bonds of sugars and water, and in the ranges 1100–1250, 1300–1350, and 1650–1700 nm, corresponding to the 3rd overtone, the combination bands, and the 1st overtone, respectively, of the C–H bonds of carotenoids. Similar absorption bands were reported in the studies of apricot, tomato, and passionfruit [42,43,44]. In the spectral interval near the red (1600–1700 nm), the bands of chlorophyll (related to the –CH2/–CH3 1st and 2nd overtones) are hidden. In the space created by the first three PCs, component analysis (CA) has highlighted the presence of clusters (Figure 2).

Along the PC1, which explains 66.74% of the variance, two clusters are clearly shown, corresponding to the year of sampling. In turn, the year of sampling is closely related to the weather, i.e., rainfall frequency and external temperature, parameters that are very different between 2013 and 2014 and that can be strongly influenced fruit characteristics. However, more interesting, inside the 2014 group, a separated sub-cluster corresponding to overripe samples (130%) along PC2 (9.15% of variance explained) can be clearly recognized. Spectra were surely influenced by the completely different physical-chemical characteristics of overripe watermelons in comparison with the other ripening stages. PC3 (7.81% of variance explained) and subsequent PCs did not seem relevant for sample clustering.

**Figure 3:**
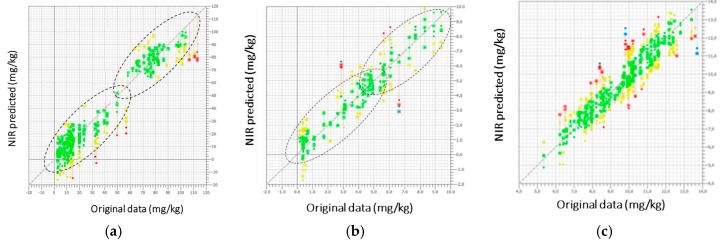
Calibration curves for lycopene (**a**), β-carotene (**b**), and total soluble solid (TSS) (**c**) content. Red and blue samples are outliers. They are visualized but excluded from the models.

This qualitative sample classification could be per se very useful for industrial applications because, by simply acquiring a spectrum, without any other chemical characterization, it is possible to know in real time if the fruit belongs to the overripe cluster (130%) and should be ruled out from the post-harvest selection line, as it would not be suitable for the market. PLS models for calibration of the three parameters of interest are shown in Figure 3.

Based on the Mahalanobis distance criterion, red and blue samples were considered outliers and eliminated from the calibration sets. For lycopene and β-carotene, data from 2013 and 2014 are almost completely separated into two different parts of the curves; in particular, samples harvested in 2013 had lower mean concentration values than samples harvested in 2014 due to the different climate conditions between the two years, cohering with the CA results. In fact, carotenoid concentration in watermelon is strongly dependent on rainfall frequency and external temperature [45]. In Italy, 2013 was characterized by particularly warm and dry weather, contrary to 2014, which will be mentioned in Italy as one of the coldest and most humid summer for decades. Different from carotenoids, TSS data are completely mixed up. TSS, which could be approximated to sugar content, depends on the ripening stage rather than on climate conditions [46]. From an NIR method development point of view, the presence of two distinct subsets for lycopene and β-carotene do not influence calibration performance, but on the contrary increase model robustness. In fact, the largest and best distributed is the database collected, the most valuable will be the source of all kinds of variations included in the models. To obtain a satisfactory prediction ability of calibration models, the data set concentration range must be well distributed and chosen to be the widest as possible to be representative. Moreover, the largest are the calibration limits, also including extreme values that rarely occur (i.e., data from particularly dry and warm seasons and particularly wet and cold summer, such as here), the most accurate will be NIR future prediction in routine analysis, because most samples will fall in the middle of the concentration interval.

**Figure 4:**
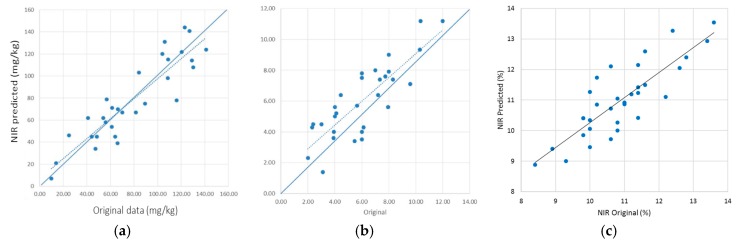
External validation plots using 35 new independent samples of watermelons for lycopene (**a**), β-carotene (**b**), and TSS (**c**).

External validations were then carried out by acquiring new independent samples during the 2015 campaign. In terms of method development, external validations evaluate the correlation between the NIR predicted data of the concentration of the parameters of interest and the corresponding values obtained with reference assays. Figure 4 shows the results obtained for lycopene, β-carotene, and TSS predictions, respectively.

Performance was evaluated plotting the NIR predicted data and the reference analysis for lycopene, β-carotene, and TSS from 35 randomly harvested samples and based on a single spectrum acquired when fruits passed along the conveyor belt. Statistical parameters of calibrations, cross-validations, and external validations were reported and are summarized in Table 1. Calibration models were selected based on the lowest PRESS values, corresponding to 11 PLS factors for lycopene and β-carotene calibrations and 8 for TSS, respectively.

**Figure 5:**
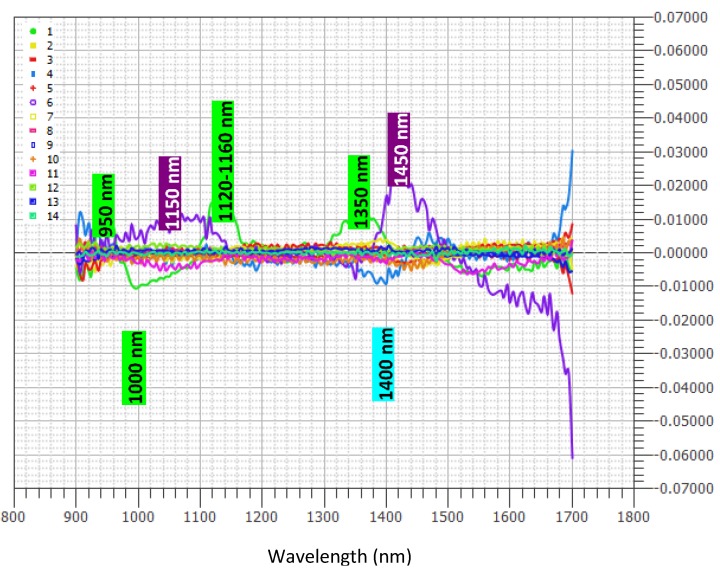
Loading plot obtained by partial least squares (PLS) applied to near-infrared (NIR) spectra.

The loadings indicated wavelengths responsible for the specific features that appeared in the corresponding scores and contributed to the quantification of the property of interest. Figure 5 highlights which wavelengths (i.e., at 950, 1000, 1120–1160, and 1350 nm) have the strongest effect on the model and bring the most information, followed by the 4th loading at 1400 nm and the 6th at 1150 and 1450 nm, respectively.

Outliers were almost exclusively spectra collected in 2014 at the highest speed rate. This means that, for a future application in a routine analysis, the maximum rate of the conveyor belt has to be set at 2400 rpm to minimize spectral aberrations that do not allow spectrum collection and concentration predictions. Although improvable, linear correlations among the predicted and original NIR data were surprisingly good, taking into account the interference of skin components in carotenoid determinations. Previous studies have been done to evaluate the shield effect of skins for the determination of TSS. Indeed, as reported by Guthrie et al. [47], a correlation coefficient lower than expected has been found for TSS in intact melon due to barrier action determined by the fruit skin. Moreover, Dull et al. [48] confirmed that R^2^ for sliced and intact melon were 0.968 and 0.600, with SEP of 1.56 and 2.18, respectively. Similar results have been found by Flores et al. [33] comparing TSS in cut and intact watermelons, using a NIR diode array spectrometer (R^2^ = 0.92, SECV = 0.49 and R^2^ = 0.81, SECV = 0.93, respectively).

It has been reported that the maximum depth of penetration of NIR light in intact fruit with the peel varied between 0 and 7 mm, decreasing at the increase of wavelengths from 700 to 2500 nm [49]. This means that, in this case, there is a concrete possibility that NIR radiation can penetrate the skin and reach the watermelon pulp, to quantify carotenoid content, or that some skin components can be correlated with carotenoid content. This aspect surely deserves further investigations.

Overall, SEP values could be considered quite satisfactory, albeit improvable, because in all three cases, they correspond to a margin of error of about or below 20%.

### 3.2. Routine Analysis

**Figure 6:**
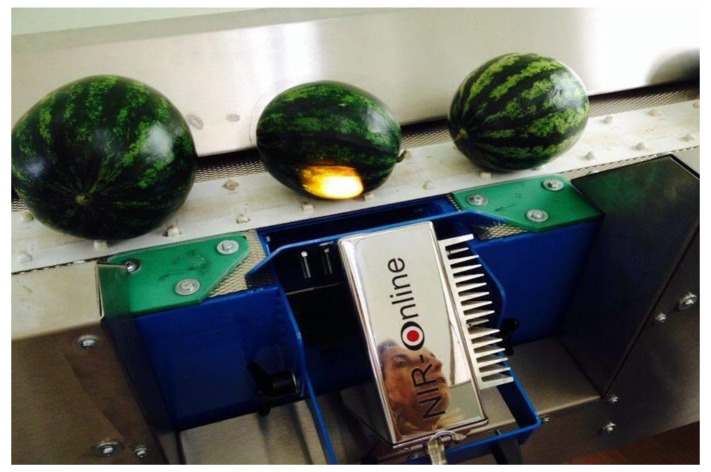
NIR in-line instrument set up on the conveyor belt system of fruit transportation of the sorting machine.

The method here developed was used to predict the concentration of TSS and carotenoids in each fruit non-destructively and in real time while passing through the sorting machine. NIR instruments have been already successfully applied for on-line and on-site applications [50,51]. In a routine analysis NIR-based system, each fruit is submitted for NIR radiation, as shown in Figure 6.

**Figure 7:**
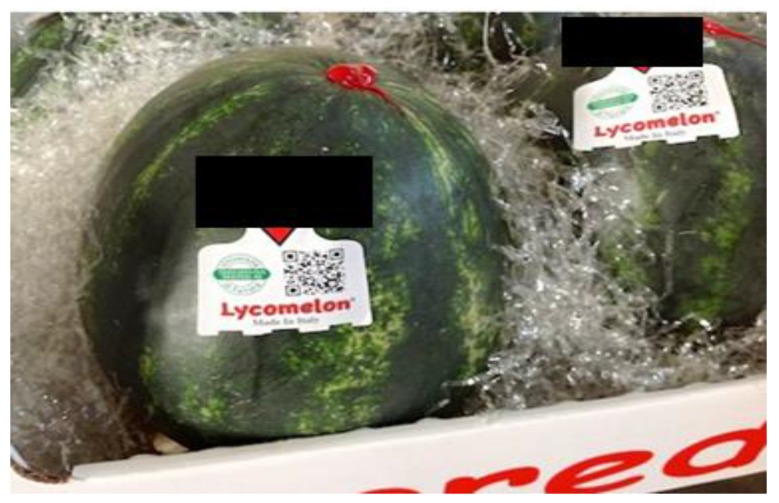
Application of the NIR method on routine analysis of watermelons ready for market.

An NIR instrument was connected to a PC-managed database where all data predicted by NIR were registered as strings corresponding to a unique sample ID. The database was progressively and automatically updated when a new sample was analyzed. At the end of the sorting machine, each fruit is then labeled with a QR-code, which permits access to the database via smartphone or tablet. By typing the sample ID, it is possible to visualize the concentration value of the fruit, together with other nutritional information (Figure 7).

In this way, based on NIR measurements, customers could check the characteristics of the fruit they are going to buy in real time—in particular, its sweetness grade (correlated to TSS, as is well known) and its lycopene and β-carotene content. In the database, measurement errors are not reported to simplify data consultation by consumers.

## 4. Conclusions

Applications of NIR on-line in post-harvest watermelon processing represent a significant step forward in improving product quality. Characterizing carotenoid content and TSS as sugar indices for each intact fruit allow farmers to valorize the nutritional value of fruits and thus increase consumers’ awareness of the beneficial health effects of fresh product consumption.

The routine analysis system, which predicts the quality of each fruit, has been in use since 2015 and can be considered a step forward in fruit valorization as a functional food.

## Figures and Tables

**Table 1:** Statistics of calibration, cross-validation, and external validation results for intact watermelons.

	Lycopene (mg/kg)		β-Carotene (mg/kg)		TTS (%)	
	CAL	EXT VAL	CAL	EXT VAL	CAL	EXT VAL
**#Sample**	100	35	100	35	100	35
**Range**	2.65–151.75	7.00–141.23	0.19–9.39	2.00–11.68	5.3–13.7	8.8–13.2
**#Spectra**	800	35	800	35	800	35
**#Outliers**	7		9		31	
**#Factors**	11		11		8	
**R^2^cal**	0.877	–	0.822	–	0.836	–
**SEC**	14.8	–	0.75	–	0.7	–
**RPD**	2.10		3.51		3.04	
**R^2^cv**	0.756	–	0.810	–	0.820	–
**SECV**	15.7	–	0.81	–	0.8	–
**R^2^_EXTVAL_**	–	0.805	–	0.737	–	0.707
**RMSEP**	–	16.2	–	0.98	–	1.4

*Subscripts*: CAL is referred to the calibration set, CV to cross validation set; EXT VAL to external validation set.
